# An Angular Radial Extended Interaction Amplifier at the W Band

**DOI:** 10.3390/s23073517

**Published:** 2023-03-28

**Authors:** Yang Dong, Shaomeng Wang, Jingyu Guo, Zhanliang Wang, Huarong Gong, Zhigang Lu, Zhaoyun Duan, Yubin Gong

**Affiliations:** National Key Laboratory of Science and Technology on Vacuum Electronics, School of Electronic Science and Engineering, University of Electronic Science and Technology of China, No. 2006 Xiyuan Avenue, High-Tech District (West District), Chengdu 611731, China

**Keywords:** AREIA, convergence angle, effective impedance, space-charge effect

## Abstract

In this paper, an angular radial extended interaction amplifier (AREIA) that consists of a pair of angular extended interaction cavities is proposed. Both the convergence angle cavity and the divergence angle cavity, which are designed for the converging beam and diverging beam, respectively, are investigated to present the potential of the proposed AREIA. They are proposed and explored to improve the beam–wave interaction capability of W-band extended interaction klystrons (EIKs). Compared to conventional radial cavities, the angular cavities have greatly decreased the ohmic loss area and increased the characteristic impedance. Compared to the sheet beam (0°) cavity, it has been found that the convergence angle cavity has a higher effective impedance and the diverging beam has a weaker space-charge effect under the same ideal electron beam area; the advantages become more obvious as the propagation distance increases. Particle-in-cell (PIC) results have shown that the diverging beam (8°) EIA performs better at an output power of 94 GHz under the condition of lossless, while the converging beam (−2°) EIA has a higher output power of 6.24 kW under the conditions of ohmic loss, an input power of 0.5 W, and an ideal electron beam of 20.5 kV and 1.5 A. When the loss increases and the beam current decreases, the output power of the −2° EIA can be improved by nearly 30% compared to the 0° EIA, and the −2° EIA has a greatly improved beam–wave interaction capacity than conventional EIAs under those conditions. In addition, an angular radial electron gun is designed.

## 1. Introduction

Radial vacuum electron devices (RVEDs) were first proposed by Arman in 1994, in which the electron beam diverged along the radial direction. Additionally, he also researched radial oscillators [1] and accelerators [2], which can obtain high power in the low-frequency band under relativistic conditions. In [3,4,5,6], theoretical, simulational, and experimental research was conducted on RVEDs, most of which operate under relativistic conditions and in the frequency band between the L band and Ku band. The above research shows that radial beam devices have the advantages of a low space-charge effect, a higher power capacity, and stronger beam–wave interactions compared to conventional devices.

**Figure 1:**
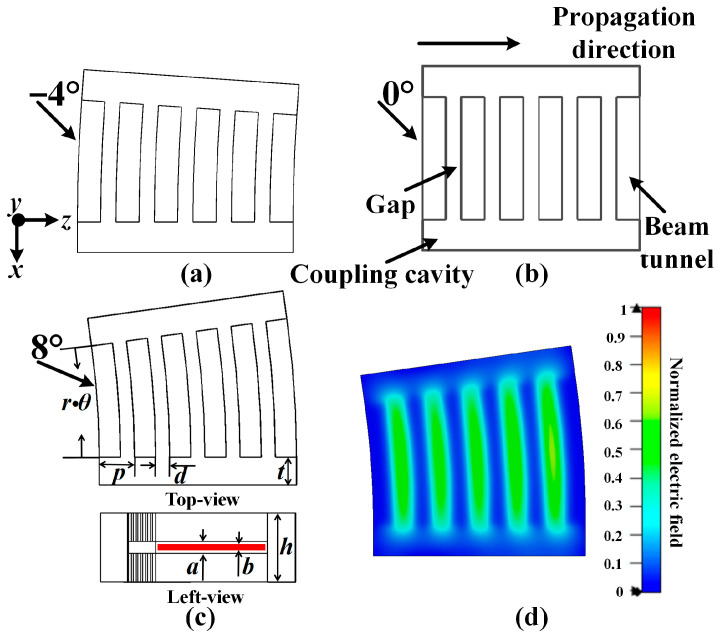
The structure of an AREIA, (**a**) −4° (convergence angle), (**b**) 0° (sheet beam), (**c**) 8° (divergence angle), (**d**) E-field distribution of the 2π mode.

However, the existing studies on radial klystron amplifiers are mainly focused on low-frequency bands and relativistic conditions. When the operating frequency is increased to the W band and the conditions are non-relativistic, the main problems are the modulation capability and ohmic loss of the radial cavity. Additionally, the radial cavity has a low *R/Q* and a large ohmic loss area. These problems can be ameliorated by the angular radial cavity, as shown in Figure 1c. The radial cavity size is greatly reduced by the divergence angle cavity. On the one hand, the characteristic impedance can be increased, and, on the other hand, the ohmic loss area is reduced.

Considering the current condition of the low-beam, the limiting of the space-charge effect is decreased, and the modulation capability becomes more important. To improve the modulation capability of the angular radial cavity in that condition, the convergence angle cavity is proposed to operate a converging beam, as shown in Figure 1a. With the propagation of the converging beam, the characteristic impedance of the corresponding convergence angle cavity can be increased.

In W-band or terahertz (THz) klystrons, the extended interaction cavity is widely used, which has the advantages of a large power capacity and a high characteristic impedance. Related research includes W-band pencil beam EIAs [7,8,9], W-band multi-beam EIAs [10], W-band sheet beam EIAs [11], G-band sheet beam EIAs [12,13], G-band pencil beam EIAs [14,15], and W-band tested EIKs [16,17,18,19]. In general, the sheet beam has a more uniform beam–wave interaction than the pencil beam, and the current density of the beam can be greatly decreased by the sheet beam. For W-band sheet beam EIAs, the relevant studies are significantly fewer than those for pencil beam EIAs.

In reference [17], the proposed EIK with three extended interaction cavities is driven by a sheet beam of 20 kV and 4 A, which can produce 7.5 kW of peak power with a beam–wave interaction efficiency of 9.38%, and it adopts a single-stage depressed collector and water cooling. In references [7,8,9], the operating currents of the pencil beam are less than 1 A, and their beam–wave interaction efficiencies are both less than 10%. To improve the beam–wave interaction capability of W-band EIKs, the AREIAs are implemented and explored.

In this paper, the diverging beam EIA and converging beam EIA will be analyzed from the perspective of beam–wave interactions and compared with the sheet beam EIA (SEIA), which can be seen as a special angular beam EIA with an angle of 0°. In Section 2, the design and dispersion of the cavities are analyzed, and the effective impedance (*M*^2^*(*R/Q*)) of the different cavities and the space-charge effect of the different beams are studied. In Section 3, the hot performance of the AREIA with three cavities is simulated. In Section 4, an angular radial electron gun of −2° is designed.

## 2. Design and Analysis

**Figure 2:**
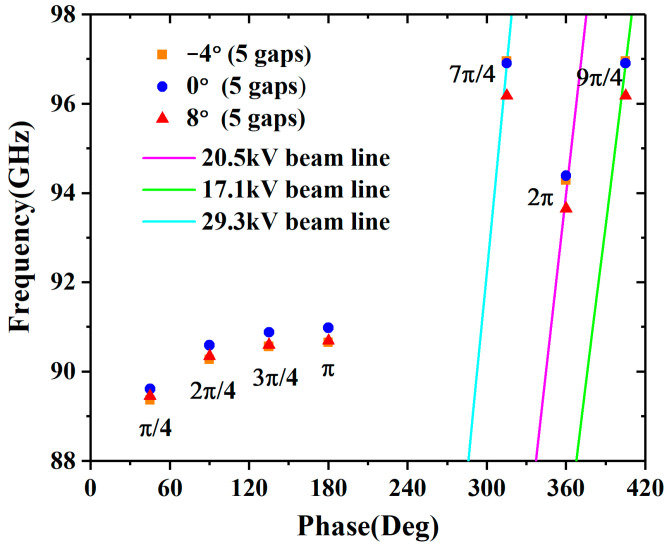
Dispersion diagrams of the different angle cavities.

The angular radial extended interaction cavities with five gaps are shown in Figure 1. As the distance of the propagation increases, the current density of the beam in the convergence angle cavity (Figure 1a) will increase, but it will decrease in the divergence angle cavity (Figure 1c). If the angle is set to zero, the angular radial cavity will turn out to be a sheet beam cavity (Figure 1b). The 2π mode (Figure 1d), which is suitable for the input and output of the signal, is chosen as the operation mode. Additionally, the optimized dimension parameters of the cavity are shown in Table 1. Figure 2 shows the dispersion diagrams of −4°, 0°, and 8° cavities with the dimensions from Table 1, and they are obtained in the CST Eigenmode Solver. The synchronous voltages of the 7π/4 and 9π/4 modes are 29.3 kV and 17.1 kV, respectively; thus, those adjacent modes will not compete with the 2π mode.

To evaluate the interaction capability of the different angle EIAs, the effects of three main parameters, i.e., *M*^2^*(*R/Q*), space-charge field, and ohmic loss, are investigated for the different angle cavities. Assuming that the cavities have the same initial arc length (2.79 mm) at Δ*r* = 0 mm during the investigation, the characteristic impedance (*R/Q*) and coupling coefficient (*M*) can be calculated as follows [14]:

(1) $$R/Q=\frac{{\left(\int_{-\infty }^{+\infty }\left|E_{z}\right|dz\right)}^{2}}{2wW_{s}},M=\frac{\int_{-\infty }^{+\infty }E_{z}e^{j\beta_{e}z}dz}{\int_{-\infty }^{+\infty }\left|E_{z}\right|dz}.$$

where *W_s_* is the total energy storage, *w* is the angular frequency, *E_z_* is the axial electric field, and *β_e_* is the propagation constant of the dc beam.

**Figure 3:**
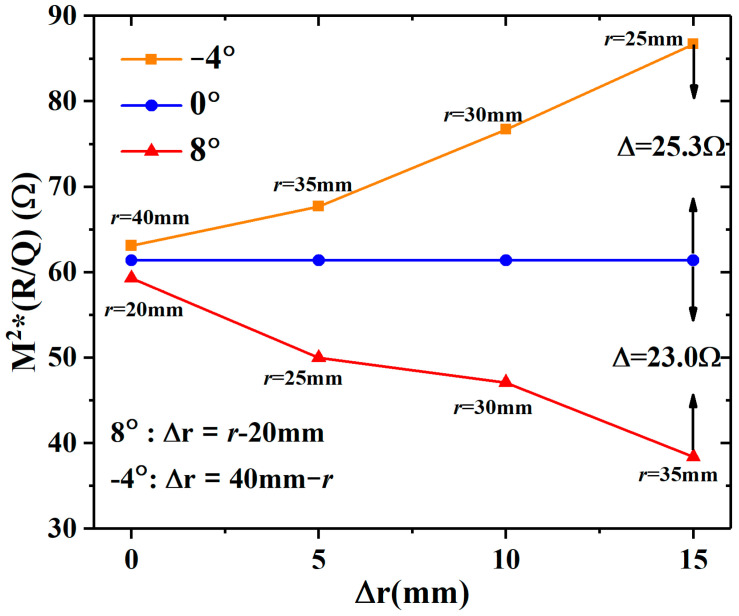
The effective impedances of the different angle cavities vary with the different initial radii in the propagation.

The higher the *M*^2^*(*R/Q*) of the cavity, the higher the degree of electron beam modulation; however, the space-charge field will prevent this process. The *M*^2^*(*R/Q*) of the −4° cavity will increase in propagation, but the 8° cavity does the opposite, as shown in Figure 3. When Δ*r* = 0 mm, there is little difference in *M*^2^*(*R/Q*) between the −4°, 0°, and 8° cavities, which means that they have almost the same modulation capability in the input cavity. In summary, the convergence angle cavity has stronger modulation capability than other cavities, and the advantage will increase as the propagation distance increases.

In the Cartesian coordinate system, Green’s function in the region of [−*l*/2, *l*/2] × [−a/2, a/2] × (−∞, +∞) can be written as follows [20]:

(2) $$\begin{array}{l} G\left(M,M_{0}\right)=\frac{2}{al\varepsilon_{0}}\sum_{m=0}^{\infty }\sum_{n=0}^{\infty }\frac{e^{-k_{z}\left|z-z_{0}\right|}}{k_{z}}\cos\left(k_{x}x\right)\cos\left(k_{x}x_{0}\right)\cos\left(k_{y}y\right)\cos\left(k_{y}y_{0}\right),\\ k_{x}=\frac{\left(2n+1\right)\pi }{l},k_{y}=\frac{\left(2m+1\right)\pi }{a},k_{z}^{2}=k_{x}^{2}+k_{y}^{2}. \end{array}$$

In the discrete particle case, the charge of each particle is −*q* (*q* = *I*/*f*/*N*, where *I* is the beam current and *N* is the number of particles in one period), and then the axial space-charge field of each particle can be calculated as follows:

(3) $$\begin{array}{ll} \left|E_{scz}(M_{0})\right| & =\left|\frac{\partial }{\partial z}\sum_{i=1}^{N}qG(M_{i},M_{0})\right|\\ & =\frac{2q}{al\varepsilon_{0}}\sum_{i=1}^{N}\sum_{m=0}^{\infty }\sum_{n=0}^{\infty }e^{-k_{z}\left|z_{i}-z_{0}\right|}\cos\left(k_{x}x_{i}\right)\cos\left(k_{x}x_{0}\right)\cos\left(k_{y}y_{i}\right)\cos\left(k_{y}y_{0}\right). \end{array}$$

It can be seen that |*Escz*| is positively correlated with cos(*k_x_x_i_*) and inversely correlated with |*z_i_*−*z_0_*|. The diverging beam has a larger range of |*x_i_*|, but the converging beam does the opposite, which means that the former has the advantage of a weaker space-charge effect. Since |*z_i_*−*z_0_*| depends on the degree of bunching, the stronger the bunching, the stronger the space-charge effect.

Table 2 lists the variations of *Q_0_* ($Q_{0}=wW_{s}/P_{loss}$, where *P_loss_* is the loss power) for the −4°, 0°, and 8° cavities with different conductivities, and there is little difference between the cavities at the same conductivity. This indicates that the ohmic loss power differs slightly between them under the same energy storage conditions. The ohmic loss power can also be written as follows:

(4) $$P_{loss}=\iint_{S}\frac{1}{2}{\left|J_{s}\right|}^{2}R_{s}dS=\iint_{S}\frac{1}{2}{\left|J_{s}\right|}^{2}\sqrt{\frac{\pi \mu f}{\sigma }}dS.$$

where *J_s_* is the surface current density, *μ* is the magnetic permeability, and *σ* denotes the conductivity. It can be found that the loss is related to the interaction area, surface current density, operating frequency, and conductivity. With the same surface current density, the divergence angle cavity will lose more power than the convergence angle cavity.

## 3. Simulation Performance

**Figure 4:**
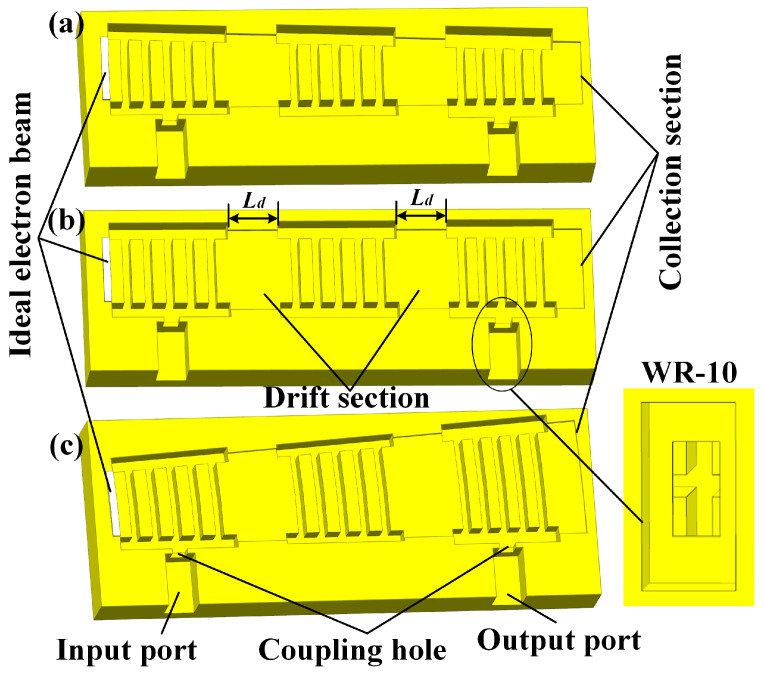
A schematic diagram (half-metal model) of the three types of EIAs: (**a**) −2°, (**b**) 0°, and (**c**) 4°.

Figure 4 shows the metal models of three different angle EIAs, all with three five-gap cavities; the standard waveguide WR-10 (2.54 mm × 1.27 mm) is used as the input and output port. A PIC simulation is conducted by CST Particle Studio; a uniform hexahedral mesh is used (the number of cells for the −2° AREIA is 2,147,740), and the boundary conditions are all set as ideal electrical boundaries.

For the comparison, three measures are adopted. First, although the ideal beams of the three EIAs are different, they have the same ideal beam area. The ideal beam current density (269 A/cm^2^) of each EIA is set to the same, and a radial focusing magnetic field of 0.8 T is used to ensure that all of the AREIAs can be focused. Second, the input cavity has the same *Q_e_*, and the coupling hole of the output cavity is optimized for the optimal output power of each EIA. Third, since the loading of the electron beam will affect the resonant frequency, the frequency of each cavity has been fine-tuned to work best at 94 GHz by adjusting *t*. Additionally, the drift distance (*L_d_* = 2.07 mm) between the cavities and the total interaction length (20 mm) of the different EIAs are the same, and the period length of the output cavity (*p_out_*) is set to 0.80 mm; thus, the electron beam can deliver more energy.

**Figure 5:**
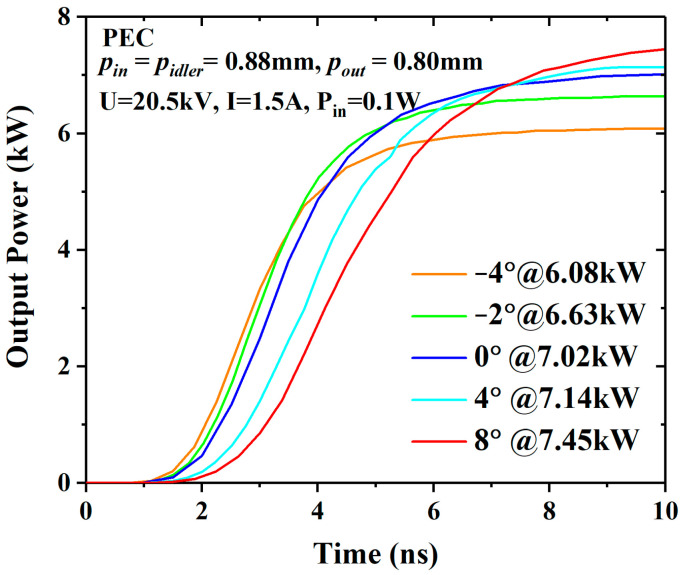
The output powers of the different EIAs in the case of lossless.

**Figure 6:**
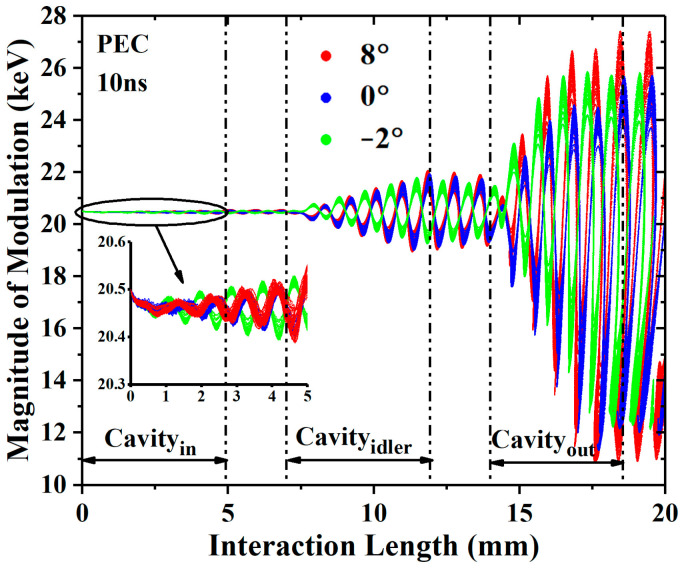
The phase space diagrams of the −2°, 0°, and 8° EIAs in the case of lossless.

For the PEC (perfect electrical conductor) shielding case, the voltage and current of the electron beam are 20.5 kV and 1.5 A, respectively, and the input power is 0.1 W. The PIC results are shown in Figure 5, and the output power increases with the angle. When *θ* = 8°, the output power of 7.45 kW increased by 6.1% compared with the 0° EIA. The modulations of the input cavities are almost no different, as shown in Figure 6. In the output cavity, the modulation of the 8° EIA is stronger than that of the 0° EIA and −2° EIA, whether in the acceleration zone or the deceleration zone. This indicates that the diverging beam EIA has taken advantage of the weaker space-charge effect, but the advantage is not great under these conditions.

**Figure 7:**
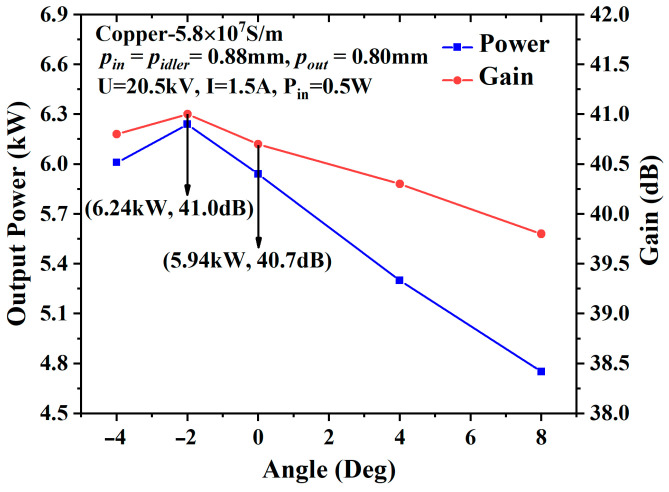
The output power and gain of the different angles in the case of loss.

For the lossy metal shielding case, the metal material is set as copper, with a conductivity of 5.8 × 10^7^ S/m, and the input power is set to 0.5 W. The output power and gain reach their maximum values (6.24 kW and 41.0 dB, respectively) at −2°, as shown in Figure 7, and the output power increases by 5.1% compared with the 0° EIA. The output power of the −4° EIA is lower than that of the −2° EIA due to the stronger space-charge effect. Instead, the output power of the diverging beam EIA drops due to the larger loss area, although it has taken advantage of the weaker space-charge effect.

**Figure 8:**
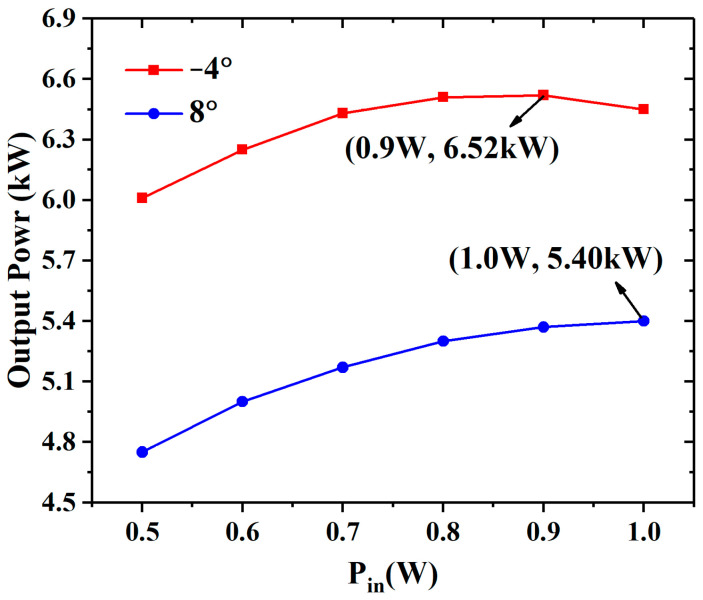
The output power of the different EIAs varies with the input power in the case of loss.

**Figure 9:**
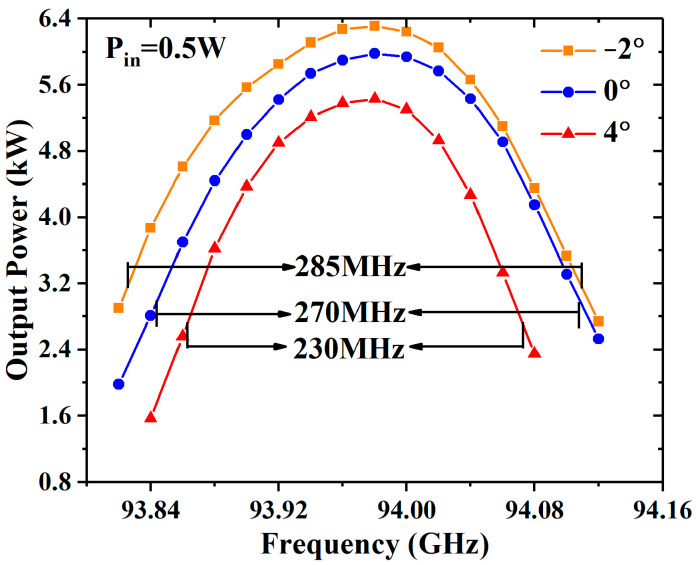
The output power of the different EIAs varies with the frequency in the case of loss.

The output powers of the −2°, 0°, and 4° EIAs with the different input powers are shown in Figure 8, and the saturated output powers are 6.59 kW, 6.06 kW, and 5.64 kW, respectively. The saturated output power of the −2° EIA increases by 8.7% compared with the 0° EIA. Figure 9 shows that the output power of the −2° EIA, 0° EIA, and 4° EIA varies with the frequency under the input power of 0.5 W, and the 3 dB bandwidths are 285 MHz, 270 MHz, and 230 MHz, respectively. The 3 dB bandwidth has little difference between the −2° EIA and 0° EIA, but the difference is up to 55 MHz between the −2° EIA and 4° EIA. The reason for this is that when the operating frequency deviates, the interaction degree decreases and the space-charge restriction effect is weakened, meaning that the stronger cavity modulation makes the −2° EIA bandwidth wider than that of the 4° EIA under the same interaction length and input power.

The influence of loss on the output power is further analyzed in Table 3. When σ decreases from 5.8 × 10^7^ S/m to 2.2 × 10^7^ S/m, the differences in the output power between the −2° EIA and the 0° EIA are 0.3 kW, 0.3 kW, and 0.37 kW. Additionally, the difference between the 0° EIA and 4° EIA is more obvious.

**Figure 10:**
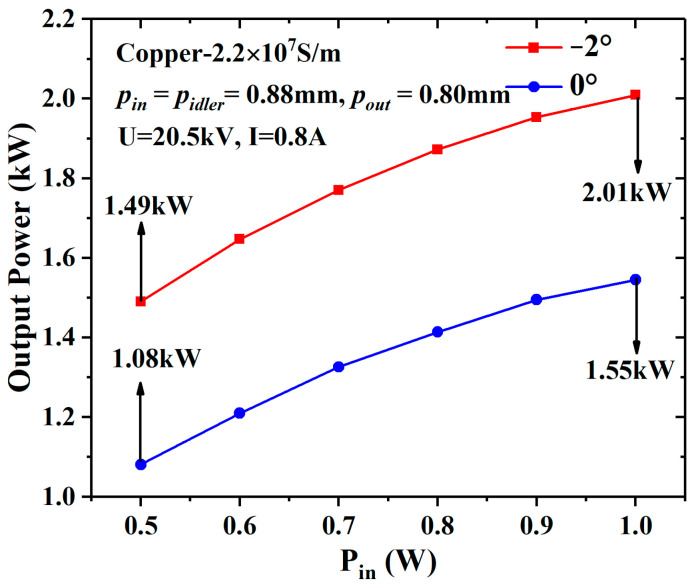
The output power of the different EIAs varies with the input power when the current is 0.8 A and the conductivity is 2.2 × 10^7^ S/m.

When the beam current decreases to 0.8 A (an ideal beam current density of 143 A/cm^2^), the space-charge effect is further depressed. As the input power increases from 0.5 W to 1 W, the difference in the output power between the −2° EIA and the 0° EIA increases from 0.41 kW to 0.46 kW, as shown in Figure 10. When the input power is 1 W, the output power of the −2° EIA increases by 29.7% compared with the 0° EIA.

Compared to previous studies [7,9,11,17], the sheet beam EIAs have a higher beam–wave interaction efficiency than the pencil beam EIAs, and the −2° EIA has better interaction performance, as shown in Table 4. Even under the high loss condition, the beam–wave interaction efficiency of the −2° EIA reached 12.3%, which is a 2.8% increase compared with 0° EIA.

**Figure 11:**
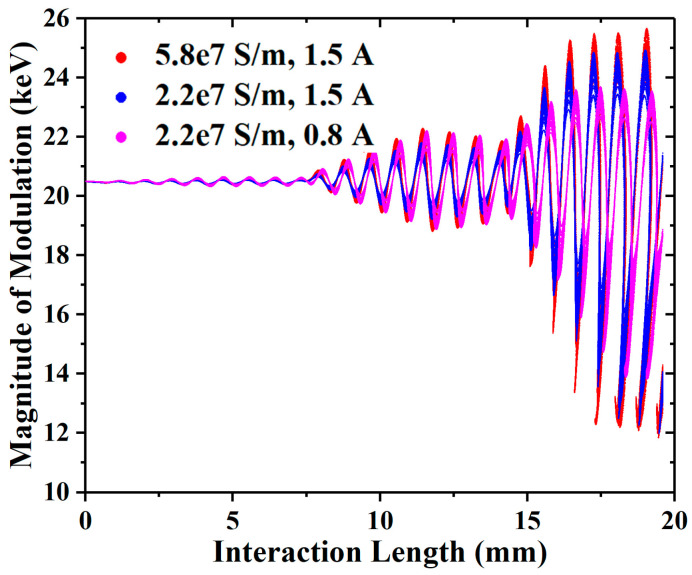
The phase space diagrams of the −2° EIA with different conductivities and beam currents.

In Figure 11, the magnitude of modulation will drop with the decrease in conductivity and beam current, especially the beam current. As it can be seen from Table 3 and Figure 11, with the increase in the loss or decrease in the beam current, the bunching is decreased, which means that the charge density in the bunching center is reduced. Additionally, the space-charge effect is depressed, as known from (3); thus, the converging beam EIA has a greater advantage. Since the balance between the space-charge effect and the cavity modulation changes with the operating conditions and structure dimensions, the optimal angle also changes.

## 4. Design of the Angular Radial Electron Gun

**Figure 12:**
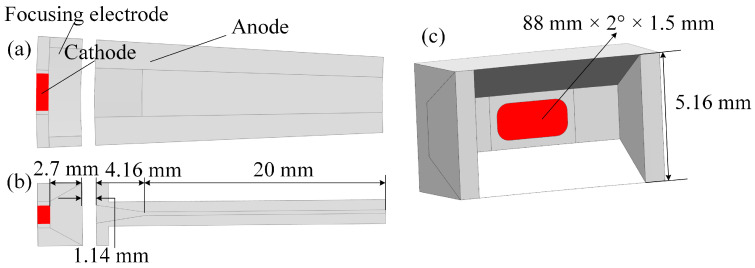
A schematic diagram of the −2° electron gun: (**a**) x–z plane cross section; (**b**) y–z plane cross section; (**c**) focusing electrode and cathode.

**Figure 13:**
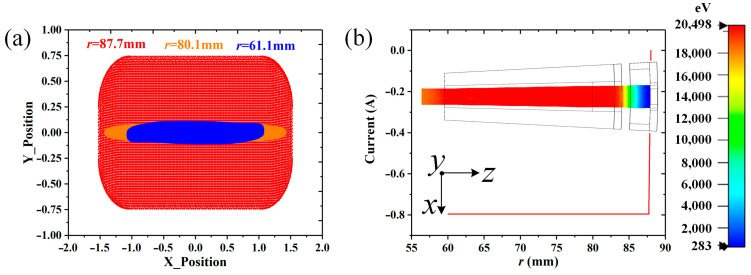
(**a**) The cross-sectional distribution of the particles, and (**b**) the total emission current along the propagation.

The divergence angle electron optical system has been designed in [21], and Figure 12 shows the sketch of the −2° angular radial electron gun. In this case, the −2° AREIA has an advantage; the beam voltage and the current are set to 20.5 kV and 0.8 A (a current density of 143 A/cm^2^), respectively. Considering the self-compression effect of the converging beam, the main requirement is to compress the beam thickness, as shown in Figure 12c. A beam size in the beam–wave entrance of 80 mm × 2° × 0.2 mm, a cathode emission area of 88 mm × 2° × 1.5 mm (a corresponding emission current density of 17.4 A/cm^2^), and an external radial focusing magnetic field of 0.5 T are added in the beam–wave section. Figure 13 shows that the beam can be compressed well under self-compression, which is obtained in the CST Particle Tracking Solver. The compressed beam size meets the requirements at the beam–wave interaction entrance, as shown in Figure 13a. Additionally, in Figure 13b, the beam transmission is 100%.

## 5. Conclusions

In our previous work [22], a 0.14 THz angular extended interaction oscillator with a diverging beam was studied and verified. The fabricated angular cavity had little machining error, which is also suitable for the processing of the −2° cavity. In this paper, the convergence angle cavity and divergence angle cavity are proposed to operate the converging beam EIA and diverging beam EIA, respectively.

The convergence angle cavity is proved to have a stronger modulation capability than the sheet beam cavity, and the diverging beam has a weaker space-charge effect than the sheet beam. The diverging beam EIA can have an advantage in output power under stronger beam–wave interactions; in the case of lossy metals, the −2° EIA manages to provide the maximum output power of 94 GHz, and the advantage becomes more obvious as the beam–wave interaction degree decreases, which is affected by the ohmic loss and beam current. The detailed research above can provide an important reference for the study of angular radial devices, such as angular radial traveling-wave tubes and angular radial backward-wave tubes.

The concept of the converging beam EIA can be further developed at the W band or the THz band. The π mode operation has been used in sheet beam EIAs [13] and pencil beam EIAs [8,14], and the angular radial beam is also suitable for π mode operations. The angular radial beam EIA is suitable for angular radial integration, which can be used for multi-beam operations. In future work, processing and cold tests will be conducted. The divergence angle electron optical system has been designed in reference [21], and the design and assembly of convergence angle electron optical systems require further research and implementation.

## Figures and Tables

**Table 1:** AREIA dimensions.

Symbol	Quantity	Dimension (mm)
*r*	Initial radius	20|40|40
*θ*	Angular angle	8°|4°|−4°
*p*	Length of period	0.88
*d*	Gap width	0.35
*a*	Beam tunnel thickness	0.30
*b*	Electron beam thickness	0.20
*h*	Height of gap	1.72
*t*	Coupling cavity width	0.70

**Table 2:** *Q_0_* with different conductivities.

σ (×10^7^ S/m)	−4° (*r* = 25 mm)	0°	8° (*r* = 35 mm)
5.8	1315	1184	1253
3.6	1036	933	987
2.2	810	729	771

**Table 3:** Output powers with different conductivities.

σ (×10^7^ S/m)	−2° EIA (kW)	0° EIA (kW)	4° EIA (kW)
5.8	6.24	5.94	5.30
3.6	5.46	5.16	4.41
2.2	4.42	4.05	3.28

**Table 4:** Comparison of the different EIAs.

Type	Operating Parameters	Output Power & Efficiency
Pencil beam EIA [7]	5 kV, 0.2 A, 5.8 × 10^7^ S/m	67 W, 6.7%
Pencil beam EIA [9]	16 kV, 0.6 A, 5.8 × 10^7^ S/m	0.9 kW, 9.4%
Sheet beam EIA [11]	75 kV, 4 A, 5.8 × 10^7^ S/m	50 kW, 16.7%
Sheet beam EIA [17]	20 kV, 4 A	7.5 kW (peak, tested), 9.4%
−2° EIA	20.5 kV, 1.5 A, 5.8 × 10^7^ S/m	6.59 kW, 21.4%
−2° EIA	20.5 kV, 0.8 A, 2.2 × 10^7^ S/m	2.01 kW, 12.3%
0° EIA	20.5 kV, 0.8 A, 2.2 × 10^7^ S/m	1.55 kW, 9.5%

## Data Availability

Data sharing is not applicable.
